# Effects of corticosterone within the hypothalamic arcuate nucleus on food intake and body weight in male rats

**DOI:** 10.1016/j.molmet.2020.02.015

**Published:** 2020-03-06

**Authors:** Chioma Izzi-Engbeaya, Yue Ma, Niki W. Buckley, Risheka Ratnasabapathy, Errol Richardson, John R. Counsell, Isabel Fernandes-Freitas, Mariana Norton, Gala Farooq, Zainab Mirza, Mingzhu Cai, Sharon Cheetham, Jonathan Seckl, Kevin Murphy, Waljit S. Dhillo, James Gardiner

**Affiliations:** 1 Section of Endocrinology and Investigative Medicine, Division of Diabetes, Endocrinology and Metabolism, Department of Medicine, Imperial College London, London, W12 0NN, UK; 2 RenaSci Ltd, BioCity, Pennyfoot Street, Nottingham, NG1 1GF, UK; 3 Endocrinology Unit, Centre for Cardiovascular Science, Queen's Medical Research Institute, 47 Little France Crescent, Edinburgh, EH16 4TJ, UK

**Keywords:** 11βHSD1, Corticosterone, Glucocorticoids, Obesity, Food intake, Body weight

## Abstract

**Objective:**

Obesity is a major cause of morbidity and mortality. Few weight-reducing medications are available, and these have limited efficacy. Cushing's Syndrome (caused by elevated glucocorticoid levels) and obesity have similar metabolic features. Though circulating glucocorticoid levels are not elevated in obesity, tissue-specific glucocorticoid levels have been implicated in the development of the metabolic phenotype of obesity. Tissue glucocorticoid levels are regulated by 11β-hydroxysteroid dehydrogenase type1 (11βHSD1), which increases the local concentration of active glucocorticoids by the production of corticosterone from 11-dehydrocorticosterone. 11βHSD1 is expressed in the hypothalamic arcuate nucleus (ARC), a major weight and appetite-regulating centre, and therefore represents a target for novel anti-obesity therapeutic agents. Thus, we sought to investigate the effect of chronic alterations of ARC corticosterone levels (mediated by 11βHSD1) on food intake and body weight in adult male rats.

**Methods:**

Recombinant adeno-associated virus particles bearing sense 11βHSD1 (rAAV-S11βHSD1) and small interfering 11βHSD1 (rAAV-si11βHSD1), respectively, were stereotactically injected into the ARC (bilaterally) of adult male Wistar rats. rAAV-GFP was injected into control groups of male Wistar rats. Food intake and body weight were measured three times a week for 70 days. Terminal brain, plasma and intrascapular brown adipose tissue (iBAT) samples were taken for measurement of mRNA expression and hormone levels.

**Results:**

Compared to controls, rAAV-S11βHSD1 injection resulted in higher ARC corticosterone levels, hyperphagia and increased weight gain. Conversely, rAAV-si11βHSD1 injection (compared to controls) resulted in lower ARC corticosterone levels, higher iBAT uncoupling protein-1 mRNA expression and less weight gain despite similar food intake.

**Conclusions:**

Therefore ARC corticosterone, regulated by 11βHSD1, may play a role in food intake and body weight regulation. These data have important implications for the development of centrally-acting 11βHSD1 inhibitors, which are currently being developed for the treatment of obesity, metabolic disorders, and other conditions.

## Highlights

• Intra-arcuate nucleus (ARC) rAAV injections altered local corticosterone levels.
• Elevated ARC corticosterone results in increased food intake and body weight.
• Lower ARC corticosterone results in lower body weight gain with similar food intake.
• Thus, ARC corticosterone may affect energy homeostasis in adult male rats.

## Graphical abstract

**Figure:**
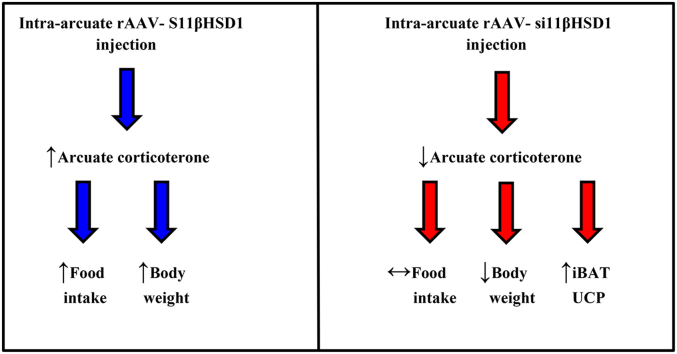

## 1 Introduction

Bariatric surgery is the most effective treatment for obesity [1], but it is not universally available and has associated risks. Therefore diet, exercise and behavioural modifications remain the mainstay of treatment with only a few licenced medicines currently available [2]. Thus, there is an urgent need to develop safe and effective medications for the treatment of obesity.

Elevated circulating glucocorticoid levels result in Cushing's Syndrome, which has many of the metabolic features seen in obesity, including elevated body weight, increased adiposity and insulin resistance. However, circulating glucocorticoid levels are not elevated in non-Cushing's Syndrome obesity [3]. Glucocorticoids exist in active (corticosterone in rodents and cortisol in humans) and inactive forms (11-dehydrocorticosterone in rodents and cortisone in humans) and only active glucocorticoids can activate the glucocorticoid receptor. In vivo, 11β-hydroxysteroid dehydrogenase type1 (11βHSD1) catalyses the conversion of inactive glucocorticoids to active glucocorticoids [4]. In adults, 11βHSD1 is highly expressed in metabolic tissues, including the brain [4].

In obesity, adipose tissue 11βHSD1 activity is elevated while hepatic 11βHSD1 activity is reduced [5]. Selective overexpression of 11βHSD1 in adipose tissue results in hyperphagia, obesity, and insulin resistance in mice [6]. However, hepatic 11βHSD1 knockout mice have improved glucose tolerance despite similar food intake and body weight to controls [7]. Therefore tissue-specific levels of glucocorticoids may play a role in the pathophysiology of obesity. Interestingly, mice with deletion of 11βHSD1 in the brain have increased food intake on a high fat diet compared to controls, although there is no difference in body weight [8]. However, it is not known which brain region is responsible for these effects, as 11βHSD1 is expressed in several brain regions [9] including the hypothalamic arcuate nucleus (ARC) [10], an area important in energy homeostasis regulation [10]. We therefore sought to investigate whether localised alteration of active glucocorticoid levels in the ARC would affect energy homeostasis.

## 2 Methods

### 2.1 Recombinant adeno-associated viruses

Recombinant adeno-associated virus (rAAV; serotype 2) encoding full-length rat 11βHSD1 (rAAV-S11βHSD1), corresponding to nucleotides 1–1258 accession number NM_008288.2, was produced, isolated, and purified as previously described [11]. 0.5 μl per hemisphere of rAAV-S11βHSD1 containing 2.96 × 10^13^ genome particles/ml was injected bilaterally into the arcuate nucleus of each animal in the overexpression group.

rAAV (serotype 2) containing small interfering constructs specific for 11βHSD1 (rAAV-si11βHSD1) was purchased from Applied Biological Materials, Canada (Catalogue number iAAV06495002). The target sequences were:

38 TGGGTTACTACTATTCTACAAATGAAGAG

187 TCGGAGGAAGGGCTCCAGAAGGTGGTGTC

619 ACTCTCTGTGTCCTCGGCTTCATAGACAC

825 TTCATTACGGTCATATAACAGGGACCTAT.

Next, 0.5 μl per hemisphere of rAAV-si11βHSD1 containing 10^13^ genome copies/ml was injected bilaterally into the arcuate nucleus of each animal in the under-expression group.

Green fluorescent protein rAAV (rAAV-GFP) was purchased from Applied Biological Materials, Canada (Catalogue number iAAV01502). 0.5 μl of rAAV-GFP containing 10^13^ genome copies/ml was injected bilaterally into the arcuate nucleus of each animal in each control group and in additional animals used for immunohistochemistry.

### 2.2 Animals

Adult male Wistar rats (Charles River, UK) were individually housed and maintained under a controlled environment (temperature 21–23 °C, 12-hour light–dark cycle, with *ad libitum* access to food and water). All animal procedures were approved under the UK Home Office Animals (Scientific Procedures) Act 1986 (Project Licence no. 70/8068). Animals were block randomised into two weight-matched groups. rAAV was stereotactically injected into the ARC bilaterally as previously described [11].

### 2.3 Experimental procedures

**Figure 1:**
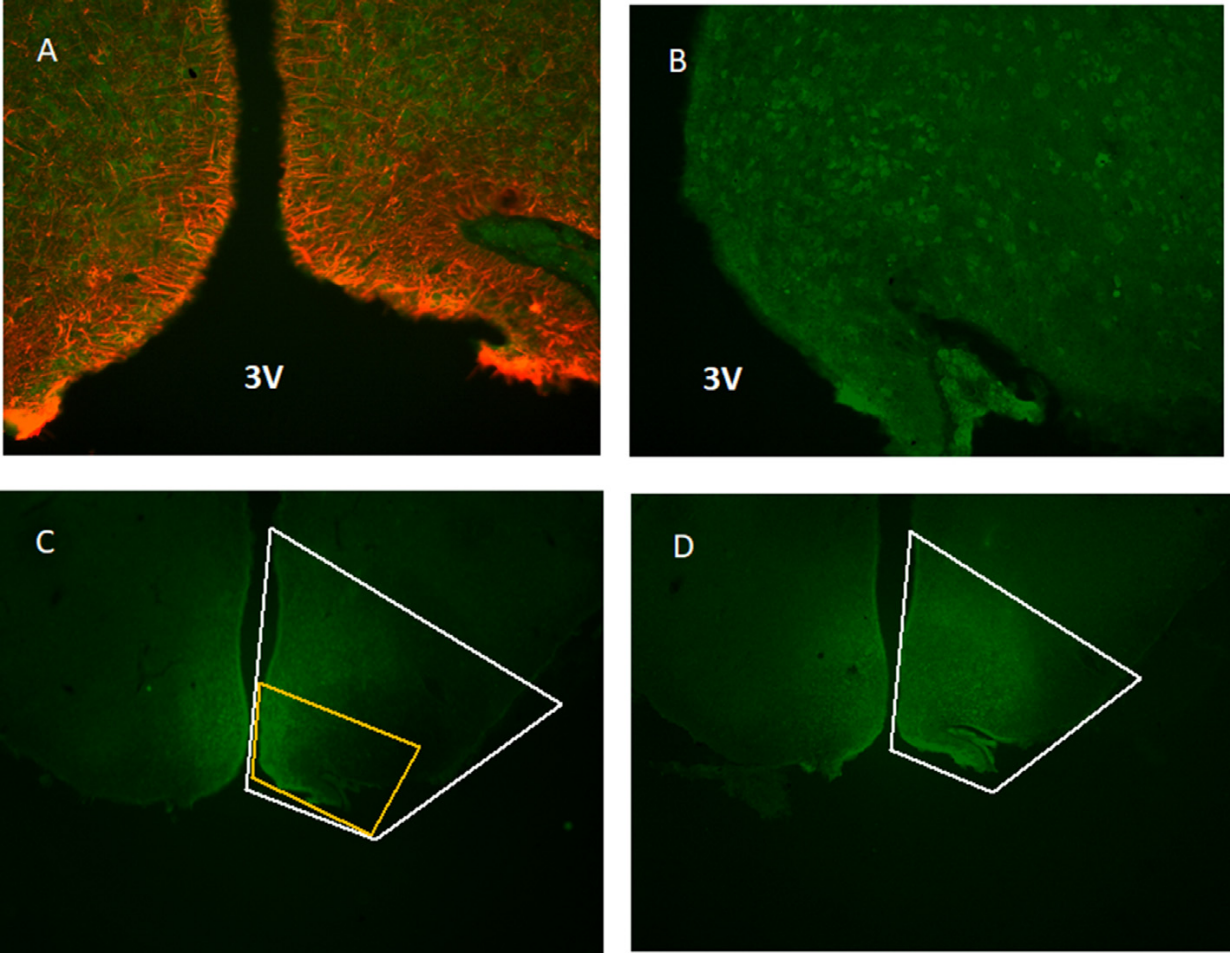
**Immunohistochemical localisation of green fluorescent protein (GFP) following rAAV injection into the arcuate nucleus. A:** Immunohistochemical detection of both GFP (green) and GFAP (red) following injection of rAAV encoding GFP into the arcuate nucleus. There is clear separation of the GFP and GFAP with no overlap of expression of the two (magnification ×20). 3V marks position of the 3rd ventricle. **B:**Immunohistochemical detection of GFP (green) following injection of rAAV encoding GFP into the arcuate nucleus (magnification ×20). 3V marks the position of the 3rd ventricle. **C:** Immunohistochemical detection of GFP (green) following injection of rAAV encoding GFP into the arcuate nucleus demonstrating extent of spread of virus, the same section is also shown in panel D (magnification ×10). The approximate extent of the arcuate is indicated by the white outline. The area indicated by the yellow outline is the approximate area shown in panels A and B. **D:** Immunohistochemical detection of GFP (green) following injection of rAAV encoding GFP into the arcuate nucleus demonstrating extent of spread of virus (magnification ×10). The approximate extent of the arcuate is indicated by the white outline. Panels B,C and D are the same section from the same animal under different magnification and/or illumination.

In experiment 1, sense 11βHSD1 rAAV (rAAV-S11βHSD1) () was injected into the S11βHSD1 group (n = 12) and green fluorescent protein rAAV (rAAV-GFP) was injected into the control group (n = 12). In experiment 2, small interfering rAAV specific for 11βHSD1 (rAAV-si11βHSD1) () was injected into the si11βHSD1 group (n = 12) and rAAV-GFP was injected into the control group (n = 12). The accuracy of intra-nuclear injection was confirmed with immunohistochemistry (detailed below) using an additional group of animals injected with green fluorescent protein rAAV (Figure 1).

The rats were killed by decapitation to minimise stress and associated elevations in brain and circulating corticosterone levels. Brains were dissected, snap frozen in dry ice-cooled isopentane and stored at −80 °C. The ARC, paraventricular nuclei (PVN), and ventromedial nuclei (VMN) were collected as previously described [11]. These nuclei were homogenised, and steroids extracted using 90% methanol in a vacuum drying centrifuge (Thermo Scientific Savant SpeedVac, Waltham, USA). Interscapular brown adipose tissue (iBAT) was dissected, snap frozen in liquid nitrogen and stored at −80 °C. RNA was extracted and measured using TaqMan qPCR (Applied Biosystems, Foster City, USA). Trunk blood was collected into tubes containing EDTA, 30 μl heparin and 200 μl aprotinin. Plasma was separated and stored at −80 °C until analysis. Carcasses were weighed and dissolved in 1 mg/g of 3M potassium hydroxide in 65% ethanol. The protein content of carcass liquid was measured with a modified Lowry protein assay (ThermoScientific, USA), and the glycerol content of carcass liquid was measured with a colorimetric assay (Randox Laboratories Ltd, UK).

### 2.4 Hormone measurement

Adenocorticotrophin (ACTH) was measured using an immunoradiometric assay (EuroDiagnostica, Netherlands) (experiment 1) or an enzyme-linked immunosorbent assay (ELISA) (MyBioSource, San Diego, USA) (experiment 2). Plasma corticosterone was measured by radioimmunoassay (MP Biomedicals, Santa Ana, USA) (experiment 1) or ELISA (Cayman Chemical, Ann Arbor, USA) (experiment 2). Brain corticosterone was measured by ELISA (Cayman Chemical, USA). The protein content of brain punch biopsies was determined using a bicinchoninic protein assay (Thermo Scientific, USA).

### 2.5 Immunohistochemistry

Four weeks after injection of rAAV-GFP, three adult male Wistar rats were killed with pentobarbitone. Transcardial perfusion with phosphate-buffered saline (PBS) followed by 4% phosphate-buffered formaldehyde was used to fix the brains. Brains were subsequently removed, equilibrated in 20% sucrose for five days, prior to snap freezing in liquid nitrogen, and stored at −80 °C. A freezing sled microtome (Shandon, UK) was used to cut 20 μm coronal sections of brain tissue, which were mounted on poly-lysine slides. Immunohistochemistry was performed utilising mouse anti-GFP antibody (1:500, ab38689). Goat anti-mouse IgG Alexa Fluor® 488 (1:500, ab150113), diluted in PBS, was used as the secondary antibody. Fluoroshield mounting medium with DAPI (ab104139) was used to fix the coverslips. The slides were then examined with a Zeiss Axiovert 100 deconvoluting microscope, using an EC Plan-Neofluar 40x/0.75 objective. Images were taken using MetaMorph software (Nashville, USA) and prepared using ImageJ 1.52t, with no adjustments made to the images.

### 2.6 Statistical analysis

Data are presented as mean ± SEM. Data from all animals were included in the analyses, apart from samples that were damaged or used for incompatible analyses. Unpaired t-tests were used for parametric data, Mann–Whitney tests were used for non-parametric data and generalized estimating equations (GEE) were used for longitudinal data. All analyses were performed using GraphPad Prism 7.0 (GraphPad Software Inc., San Diego, USA); apart from GEE, which were performed on body weight and food intake data using STATA 14.1 (Statcorp LLC, Texas, USA). Significance was set at p < 0.05.

## 3 Results

**Figure 2:**
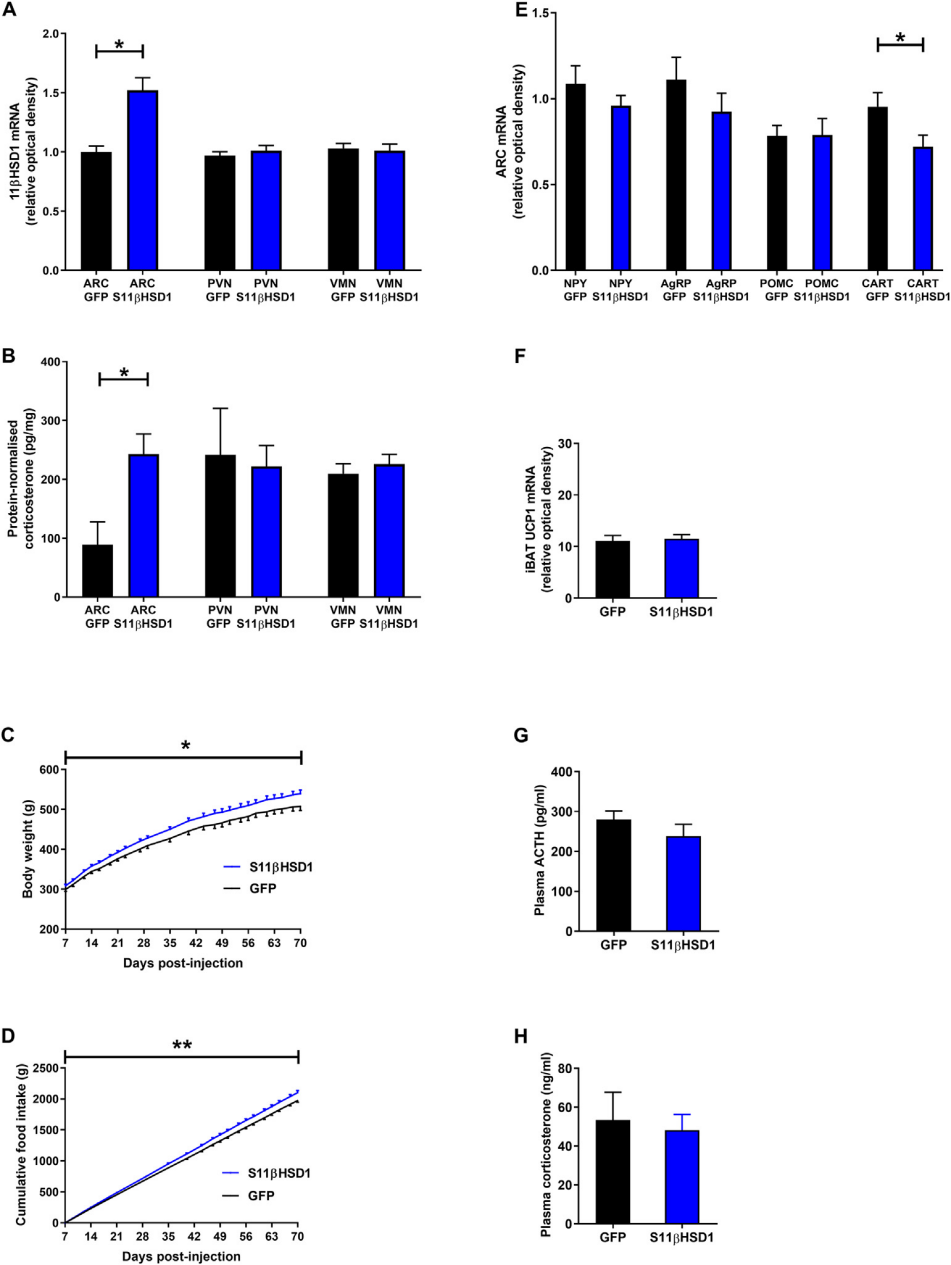
**Increased corticosterone in the arcuate nucleus of adult male rats is associated with hyperphagia and elevated body weight. A**: Bilateral injection of sense 11βHSD1 rAAV (rAAV-S11βHSD1) into the arcuate nucleus (ARC) results in a significant increase in 11βHSD1 mRNA expression within the ARC but does not affect 11βHSD1 expression in the paraventricular nucleus (PVN) or ventromedial nucleus (VMN); GFP: n = 3, S11βHSD1: n = 6; ∗p < 0.05 (Mann–Whitney test). **B**: Bilateral injection of rAAV-S11βHSD1 into the ARC results in a significant increase in corticosterone levels within the ARC but does not affect corticosterone levels in the PVN and VMN; GFP: n = 3, S11βHSD1: n = 6; ∗p < 0.05 (Mann–Whitney test). **C:** Elevated ARC corticosterone is associated with higher body weight compared to controls; GFP: n = 11, S11βHSD1: n = 12; ∗p < 0.05 (generalized estimating equation, GEE). **D:** Elevated ARC corticosterone is associated with higher food intake compared to controls; GFP: n = 11, S11βHSD1: n = 12; ∗∗p < 0.01 (GEE). **E**: Elevated ARC corticosterone is associated with reduced expression of cocaine- and amphetamine-regulated transcript (CART) in the ARC but does not affect the expression of neuropeptide-Y (NPY), Agouti-related peptide (AgRP) and pro-opiomelanocortin (POMC); GFP: n = 3, S11βHSD1: n = 6; ∗p < 0.05 (Mann–Whitney test). **F**: Elevated ARC corticosterone does not affect interscapular brown adipose tissue (iBAT) uncoupling protein-1 (UCP1) expression; GFP: n = 11, S11βHSD1: n = 12 (unpaired t-test). **G**: Elevated ARC corticosterone does not affect plasma adenocorticotrophic hormone (ACTH) levels; GFP: n = 11, S11βHSD1: n = 12 (unpaired t-test). **H**: Elevated ARC corticosterone does not affect plasma corticosterone levels; (GFP: n = 11, S11βHSD1: n = 12 (unpaired t-test).

After 70 days, the expression of 11βHSD1 was increased in the ARC of iARC-S11βHSD1 rats compared to rAAV-GFP rats, as was corticosterone. The levels of 11βHSD1 and corticosterone were unaffected in neighbouring nuclei (Figure 2A,B). Body weight (Figure 2C) and food intake (Figure 2D) were approximately 6% higher in the iARC-S11βHSD1 group compared to the iARC-GFP group. Anorexigenic cocaine- and amphetamine-regulated transcript (CART) mRNA expression was significantly lower in the ARC of iARC-S11βHSD1 rats (Figure 2E). iBAT UCP1 expression (Figure 2F) was similar in iARC-S11βHSD1 and iARC-GFP rats. Changes in hormone levels were confined to the ARC as plasma ACTH (Figure 2G) and plasma corticosterone (Figure 2H) were similar in iARC-S11βHSD1 and iARC-GFP rats. Body composition was similar in both groups (percentage fat: iARC-GFP rats 20.8 ± 1.5% vs iARC-S11βHSD1 rats 19.4 ± 1.7%, p = 0.56; percentage protein: iARC-GFP rats 18.4 ± 0.4% vs iARC-S11βHSD1 rats 18.7 ± 0.5%, p = 0.50).

**Figure 3:**
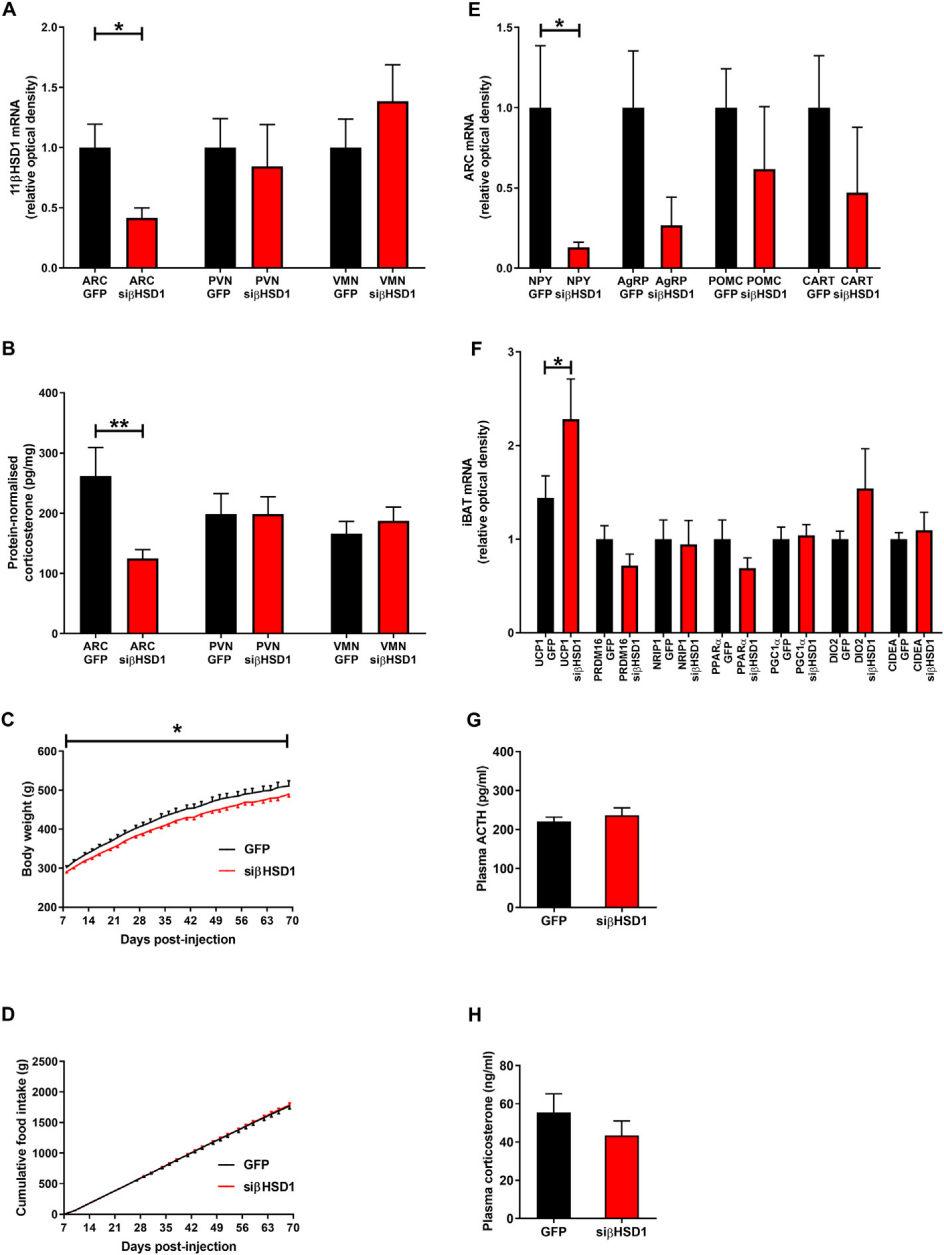
**Decreased corticosterone in the arcuate nucleus of adult male rats is associated with attenuated body weight gain. A**: Bilateral injection of small interfering rAAV specific for 11βHSD1 (rAAV-si11βHSD1) into the arcuate nucleus (ARC) results in a significant decrease in 11βHSD1 mRNA expression within the ARC but does not affect 11βHSD1 expression in the paraventricular nucleus (PVN) or ventromedial nucleus (VMN); GFP: n = 3, si11βHSD1: n = 3; ∗p < 0.05 (Mann–Whitney test). **B**: Bilateral injection of rAAV-si11βHSD1 into the ARC results in a significant reduction in corticosterone levels within the ARC but does not affect corticosterone levels within the PVN or VMN; GFP: n = 9, si11βHSD1: n = 10; ∗∗p = 0.01 (unpaired t-test). **C**: Reduced ARC corticosterone is associated with lower body weight compared to controls; GFP: n = 11, si11βHSD1: n = 10; ∗p < 0.05 (generalized estimating equation, GEE). **D:** Reduced ARC corticosterone does not affect food intake; GFP: n = 11, si11βHSD1: n = 10 (GEE). **E**: Reduced ARC corticosterone is associated with decreased expression of neuropeptide-Y (NPY) in the ARC but does not affect the expression of Agouti-related peptide (AgRP), cocaine- and amphetamine-regulated transcript (CART) or pro-opiomelanocortin (POMC); GFP: n = 3, si11βHSD1: n = 3; ∗p < 0.05 (Mann–Whitney test). **F**: Reduced ARC corticosterone is associated with increased interscapular brown adipose tissue (iBAT) uncoupling protein-1 (UCP1) expression; GFP: n = 11, si11βHSD1: n = 10; ∗p < 0.05 (unpaired t-test). There was no significant difference in mRNA expression of iBAT peptides associated with thermogenesis (i.e. PR domain containing 16 (PRDM16), nuclear receptor interacting protein 1 (NRIP1), peroxisome proliferator-activated receptor-α (PPARα), peroxisome proliferator-activated receptor gamma coactivator 1-α (PGC1α), iodothyronine deiodinase 2 (DIO2), and cell death activator (CIDEA)) in the group injected with rAAV-si11βHSD1 (siβHSD1) and the group injected with rAAV-GFP (GFP). **G**: Reduced ARC corticosterone does not affect plasma adrenocorticotrophic hormone (ACTH) levels; GFP: n = 11, si11βHSD1: n = 10 (unpaired t-test). **H**: Reduced ARC corticosterone does not affect plasma corticosterone levels; GFP: n = 9, si11βHSD1: n = 9; (unpaired t-test).

After 70 days, the expression of 11βHSD1 was decreased in the ARC of iARC-si11βHSD1 rats compared to rAAV-GFP rats, as was corticosterone. Levels of 11βHSD1 and corticosterone were unaffected in neighbouring nuclei (Figure 3A,B). Body weight was 5% lower in rAAV-si11βHSD1 rats compared with controls (Figure 3C). However, food intake was similar in both iARC-si11βHSD1 and iARC-GFP rats (Figure 3D). Orexigenic Neuropeptide Y (NPY) mRNA expression was significantly lower in the ARC of iARC-si11βHSD1 rats (Figure 3E). iBAT UCP1 mRNA expression was higher in iARC-si11βHSD1 rats compared to controls (Figure 3F). Plasma ACTH (Figure 3G) and plasma corticosterone (Figure 3H) were unaffected by intra-ARC rAAV-si11βHSD1 injection. Although iBAT UCP1 mRNA was significantly increased in the iARC-si11βHSD1 rats, no differences were detected in mRNA expression of other key iBAT genes (Figure 3F). Body composition was similar in both groups (percentage fat: iARC-GFP rats 26.7 ± 2.5% vs iARC-si11βHSD1 rats 29.0 ± 1.9%, p = 0.46; percentage protein: iARC-GFP rats 9.7 ± 0.4% vs iARC-si11βHSD1 rats 10.8 ± 0.6%, p = 0.13).

## 4 Discussion

Our results suggest that corticosterone within the ARC in adulthood may play a role in the regulation of food intake and body weight. Elevated ARC corticosterone in rats increased food intake and promoted weight gain. Conversely, decreased ARC corticosterone was associated with lower body weight gain, with no change in food intake.

Studies have demonstrated that glucocorticoids increase expression of the orexigenic neuropeptides agouti-related peptide (AgRP) [12,13] and NPY in the ARC [12], and AgRP acts centrally in vivo to reduce BAT thermogenesis [14]. Furthermore, adrenalectomy reduces expression of NPY within the ARC [15]. Interestingly, 11βHSD1 knockout mice (which had similar circulating corticosterone levels to control mice), had decreased CART expression on a low fat diet, but increased AgRP and pro-opiomelanocortin POMC expression on a high fat diet [10].

In our study, increased ARC corticosterone was associated with reduced ARC anorexigenic CART expression consistent with increased food intake in these animals. Decreased ARC corticosterone was associated with reduced ARC orexigenic NPY expression. In animals with decreased ARC corticosterone, AgRP expression was also suppressed, but this suppression was not statistically significant (which may have been due to the small sample size).

Therefore the matched food intake associated with lower body weight observed with reduced ARC corticosterone in our study cannot be fully explained by the changes in expression of the appetite-regulating neuropeptides measured. Due to the fact that neuropeptide RNA expression was measured 70 days after the intra-ARC rAAV injections, the patterns of neuropeptide expression that we detected could be due to direct responses to manipulation of 11βHSD1 expression and consequent changes in ARC corticosterone levels and/or a compensatory response to the body weight change.

As lower body weights were associated with reduced ARC corticosterone levels, brain penetrance may be required for the maximum efficacy of 11βHSD1 inhibitors. It is possible that diffusion of corticosterone into the PVN and VMN may have occurred, and this may be an explanation for our food intake findings, as these nuclei also play important roles in the regulation of food intake. However, we did not detect changes in corticosterone levels following intra-ARC rAAV injection in the PVN and VMN. Some 11βHSD1 inhibitors have been shown to be centrally-active, as following oral administration to rodents, their concentrations within the brain exceeded their respective IC50s, they significantly reduced the activity of 11βHSD1 in the brain by >80%, and reduced body weight [8]. Therefore these agents are likely to affect the ARC as well as other appetite-regulating nuclei via the modulation of central corticosterone levels; however, no information about central corticosterone levels following the administration of these 11βHSD1 inhibitors was provided [8].

iBAT UCP1 mRNA expression was elevated by decreased ARC corticosterone. As iBAT mRNA expression was measured at the end of the experiment, it is not surprising that UCP1 expression (which is important for iBAT thermogenesis) was increased, whilst genes important for BAT differentiation and expansion in response to stimuli (e.g. PRDM16) were unchanged. Furthermore, rats with reduced ARC corticosterone had lower body weight than controls despite matched food intake. Taken together, these results suggest that decreased corticosterone levels in the ARC may increase BAT-mediated energy expenditure, and thus may limit body weight gain.

ARC-specific increase in corticosterone resulted in hyperphagia on a normal chow diet; therefore reduced ARC corticosterone would have been expected to result in reduced food intake. However, reduced ARC corticosterone did not affect food intake. The complex relationship between central 11βHSD1 activity (influencing corticosterone levels) and food intake is further highlighted by a study using 11βHSD1 brain knockout mice, which reported increased food intake (compared to controls) on a high fat diet in the knockout mice [8]. Interestingly, the body weights of the animals with lower central 11βHSD1 expression were lower than the body weights of controls [8], which suggests that energy expenditure may play an important role in the regulation of body weight when 11βHSD1 activity is reduced centrally. Consistent with this finding, the expression of iBAT UCP1 mRNA (an indirect measure of energy expenditure, which is strongly and positively correlated with energy expenditure [16]) was increased in rats with lower central 11βHSD1 expression in our study. However, direct measurements of energy expenditure would be required before firm conclusions can be made about the relative contributions of food intake and energy expenditure to body weight following alterations of corticosterone levels within specific brain nuclei.

Altered ARC corticosterone did not affect systemic corticosterone and ACTH levels. Since the ARC is not the major site for negative feedback regulation of adrenal corticosterone production [17], modulation of 11βHSD1 within the ARC would not be expected to alter systemic corticosterone and ACTH levels. 11βHSD1 inhibitors have been associated with increased systemic ACTH and adrenal steroid levels in rodents and humans [[18], [19], [20]]. However, restoration of hepatic 11βHSD1 activity prevents elevation of systemic ACTH in global 11βHSD1 knockout mice [21]. We have demonstrated that intra-ARC rAAV-si11βHSD1 injection, reduced ARC corticosterone which results in less body weight gain than controls, without affecting circulating ACTH and corticosterone. Therefore the development of centrally active 11βHSD1 inhibitors, with limited hepatic activity, may produce weight loss without causing increased levels of circulating ACTH and adrenal androgens.

Our work raises some interesting future directions for research in this area. It would be interesting to test the effects of ARC corticosterone manipulation in high-fat, diet-induced obese rats as a model of obesity. In addition, it would be interesting to investigate the effects of glucocorticoids in other brain nuclei to determine their effects on energy homeostasis. Whilst we did not target other nuclei in this study, this has been studied previously [22,23]. PVN corticosterone implantation did not alter food intake in intact male rats, but it increased food intake in adrenalectomized rats [22]. The administration of corticosterone into the amygdala or prefrontal cortex of male rats did not affect food intake or body weight before or after adrenalectomy [23].

## 5 Conclusions

The alteration of corticosterone levels by the modulation of 11βHSD1 expression within the ARC is associated with corresponding changes in food intake, iBAT UCP1 expression, and body weight, without affecting circulating corticosterone and ACTH. These data are important for the ongoing development of 11βHSD1 inhibitors for the treatment of obesity, as brain penetrance may be required for the maximal metabolic efficacy of these agents.

## Author statement

**Chioma Izzi-Engbeaya**: Data curation; Formal analysis; Funding acquisition; Investigation; Methodology; Writing - original draft; Writing - review & editing. **Yue Ma**: Data curation; Formal analysis; Investigation; Methodology; Writing - original draft; Writing - review & editing. **Niki W. Buckley**: Data curation; Formal analysis; Investigation; Methodology; Writing - review & editing. **Risheka Ratnasabapath**y: Investigation; Methodology; Writing - review & editing. **Errol Richardson**: Data curation; Formal analysis; Investigation; Methodology; Writing - review & editing. **John Counsell**: Investigation; Methodology; Writing - review & editing. **Isabel Fernandes-Freita**s: Investigation; Writing - review & editing. **Mariana Norton**: Investigation; Writing - review & editing. **Gala Farooq**: Investigation; Writing - review & editing. **Zainab Mirza**: Investigation; Writing - review & editing. **Mingzhu Cai**: Investigation; Writing - review & editing. **Sharon Cheetham**: Funding acquisition; Investigation; Writing - review & editing. **Jonathan Seckl**: Investigation; Methodology; Writing - review & editing. **Kevin Murphy**: Investigation; Methodology; Writing - review & editing. **Waljit S. Dhillo**: Conceptualization; Funding acquisition; Formal analysis; Writing - original draft; Writing - review & editing. **James Gardiner**: Conceptualization; Funding acquisition; Formal analysis; Methodology; Writing - original draft; Writing - review & editing.
